# Spinal Cord Stimulation in an Elderly Patient With Severe Scoliosis and Failed Back Surgery Syndrome: A Case for Reconsidering Anatomical Contraindications

**DOI:** 10.7759/cureus.89281

**Published:** 2025-08-03

**Authors:** Shivang Patel, Jalal Ibrahim, Feross Habib, Harthik Kambhampati, Tony El-Hayek

**Affiliations:** 1 College of Medicine, Lake Erie College of Osteopathic Medicine, Bradenton, USA; 2 Interventional Pain Management, Mercy Health-Allen Hospital, Oberlin, USA

**Keywords:** anatomical contraindications, chronic low back pain (clbp), elderly patients, failed back surgery syndrome (fbss), neuromodulation therapy, spinal cord stimulation (scs)

## Abstract

Spinal cord stimulation (SCS) has demonstrated efficacy in treating intractable pain associated with failed back surgery syndrome (FBSS), though its success in patients with severe spinal deformities remains uncertain. This case report presents a 78-year-old female patient with FBSS, advanced lumbar scoliosis, and multiple prior spinal surgeries, who experienced severe, debilitating pain despite extensive conservative and pharmacological treatments. Imaging revealed significant degenerative changes, spinal subluxations, and multilevel stenosis. After a successful SCS trial with 70-80% pain relief and full restoration of activities of daily living (ADLs), a nonrechargeable SCS system (Vanta) was implanted. Despite anatomical challenges, optimized electrode placement and programming adjustments resulted in sustained pain reduction and functional improvement. This case illustrates the potential feasibility of SCS in the setting of complex spinal pathology and emphasizes the value of individualized treatment planning. While not broadly generalizable, it contributes to the growing body of evidence supporting carefully selected use of SCS in anatomically challenging patients

## Introduction

Low back pain (LBP) is one of the most disabling conditions worldwide, affecting approximately 619 million people in 2020, a 60.4% increase in prevalence from 1990 [1]. Even though diagnostic and surgical techniques have advanced, the rising rate of spinal surgery has not been matched with a relative increase in pain relief [1]. Current literature indicates that 5% to 27.6% of patients develop chronic pain following spinal surgery, with a pooled prevalence of 14.97% [1]. Patients with chronic or recurrent lumbar spinal pain after surgery are diagnosed with failed back surgery syndrome (FBSS) [2]. Usually, the pain is located near the spot of surgery. FBSS is defined by the International Association for the Study of Pain as persistent or recurrent LBP in the same anatomical region, either continuing despite spinal surgery or emerging after surgery, without a clearly identifiable cause [2]. The condition can be caused by various factors, including inappropriate decompression, scar tissue formation, altered biomechanics, or further degenerative disease progression at adjacent levels to the fusion [2]. FBSS is a highly diverse clinical syndrome that significantly impacts patient function and quality of life.

Given the high failure rates of revision spine surgeries, minimally invasive approaches are generally preferred as first-line options for pain management after conservative measures [1]. Neuromodulation involves placing an electrode in the epidural space to deliver targeted electrical impulses to the spinal cord [1]. This technique uses a spinal cord stimulation (SCS) to disrupt pain signal transmission before the signals reach the brain. This case discusses a patient who received SCS despite her age and anatomical challenges. 

SCS is relatively contraindicated in patients with complex spinal anatomy, including severe scoliosis, spinal stenosis, prior laminectomy, and degenerative kyphosis. These conditions can distort spinal dimensions, complicate epidural access, and hinder effective lead placement, especially in elderly patients with tight interlaminar spaces [3,4]. Studies have also shown that patients with adult spinal deformity (ASD) often experience reduced SCS efficacy, with some requiring surgical correction for meaningful relief [5,6]. These anatomical challenges highlight the need for careful patient selection and preoperative planning when considering SCS.

While complex spinal anatomy is relatively contraindicated, advanced age is not a contraindication to SCS, but older patients are less frequently offered the procedure. This is due to concerns about frailty, comorbidities, and perceived lower success rates [7]. Evidence on SCS outcomes in the elderly is mixed, with some studies showing comparable results to younger patients and others suggesting reduced efficacy [7-9]. This case adds to the growing body of evidence supporting the use of SCS in select elderly patients by demonstrating a successful outcome in a 78-year-old woman. Despite the common reluctance to offer SCS to older individuals, she experienced significant pain relief and functional improvement, highlighting the potential benefit of neuromodulation in appropriately selected elderly patients 

In this case, we present a 78-year-old female patient with FBSS, severe kyphoscoliosis, prior laminectomies, and multilevel degenerative spine disease who suffered from chronic, debilitating pain unresponsive to conservative and interventional treatments. Despite prior lumbar fusion and complex spinal anatomy, she achieved 70-80% pain relief and full restoration of ADLs following SCS. While SCS is commonly used for refractory back pain, it is less frequently offered to elderly patients, despite age not being a contraindication [7]. Her severe spinal deformity also posed a relative contraindication due to technical challenges. This case highlights the potential of SCS in elderly patients with complex anatomy when carefully selected and managed.

## Case presentation

A 78-year-old female patient with a previous history of FBSS (surgery listed below) presented to the pain clinic with bilateral radiation of persistent and progressive lower back pain to the posterior thighs for over two years. At the clinic, she described her pain as extremely debilitating, with a visual analog scale (VAS) score of six or higher during activity. The pain severely limits her mobility and daily functioning; she reports being unable to stand upright or walk due to its intensity.

Before her surgery, she noted that she received a one-time steroid spinal injection, which provided no relief of symptoms. In May 2022, the patient underwent a decompressive L4 lumbar laminectomy, left-sided transforaminal lumbar interbody fusion at L4-5, and posterior spinal instrumentation fusion. The surgery was done with an autogenous bone graft, and the plan at that time was to stabilize mechanical instability and spinal stenosis. The patient noted that although the surgery initially alleviated her symptoms, the symptoms recurred and were worse shortly afterward, consistent with FBSS. 

Conservative management for her FBSS with her primary care provider involved prolonged administration of tramadol 50 mg, taken twice daily as needed. She also tried various adjunct therapies such as antineuropathic medications and muscle relaxants, which did not help her. 

Her primary care doctor declined to continue her pain management, and pain management was consulted to assume long-term responsibility for her chronic pain disorder as of May 2024. Her pain remained predominantly unresponsive to pharmacologic management at the time of assessment, and she was considering interventional treatments to restore mobility and improve function.

**Figure 1:**
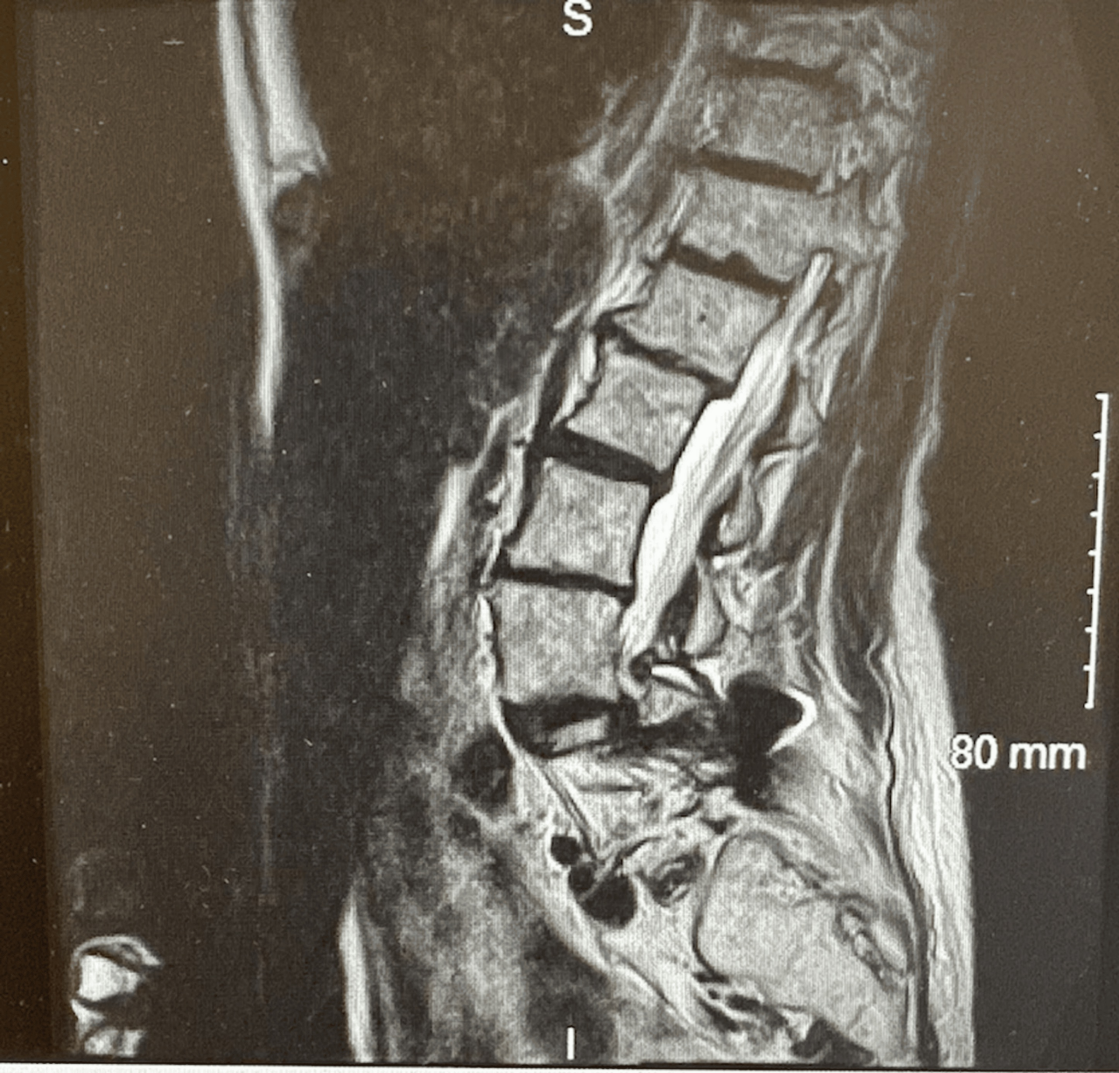
MRI of the lumbar spine MRI: magnetic resonance imaging Demonstrating multilevel severe degenerative changes in the lumbar spine

Advanced imaging was conducted to assess ongoing structural disease at the pain clinic. Magnetic resonance imaging (MRI) of the lumbar spine demonstrated multilevel severe degenerative changes (Figure 1). She was unable to tolerate the time needed for an accurate MRI due to her pain.

**Figure 2:**
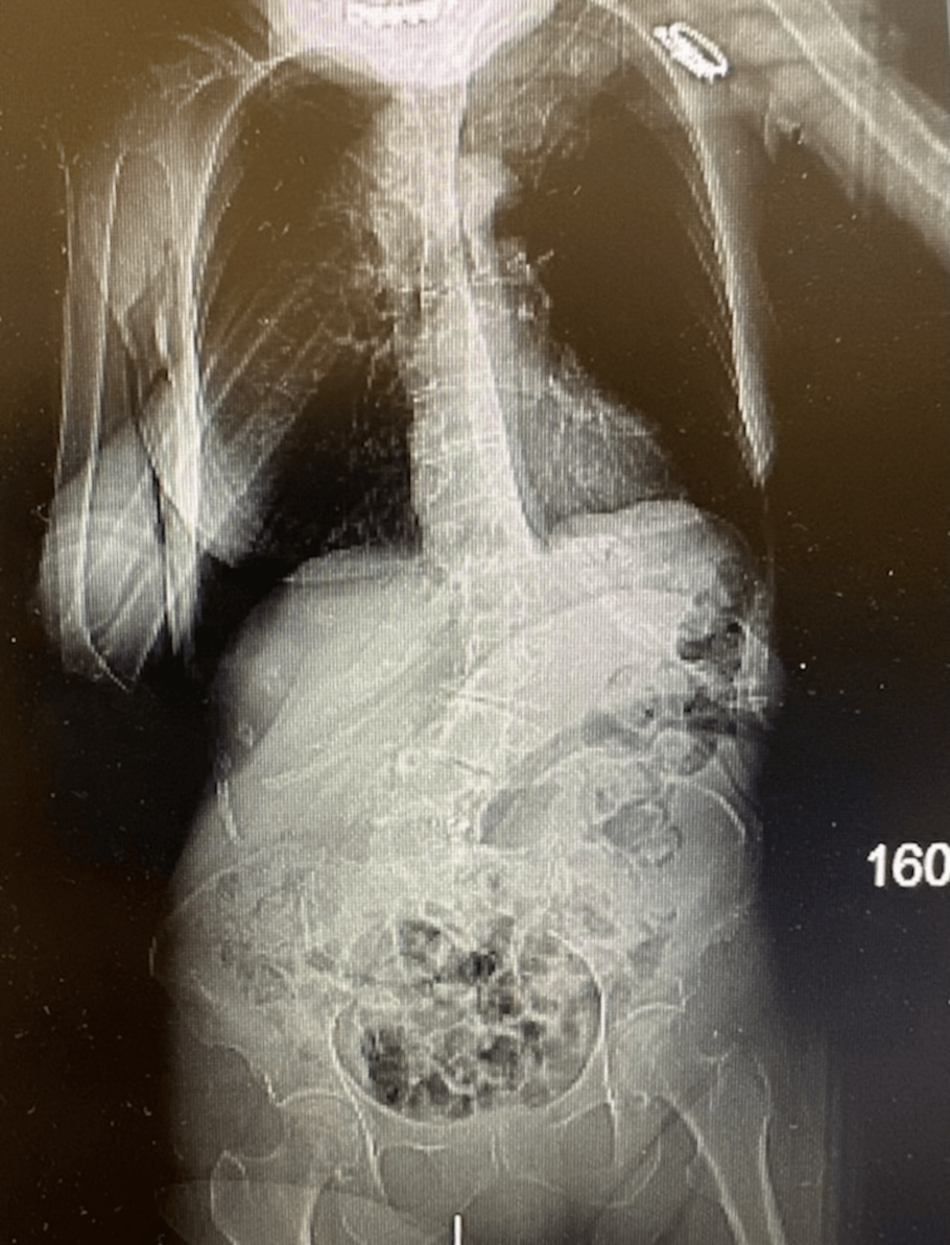
Coronal view CT scan of thoracolumbar spine CT: computed tomography

**Figure 3:**
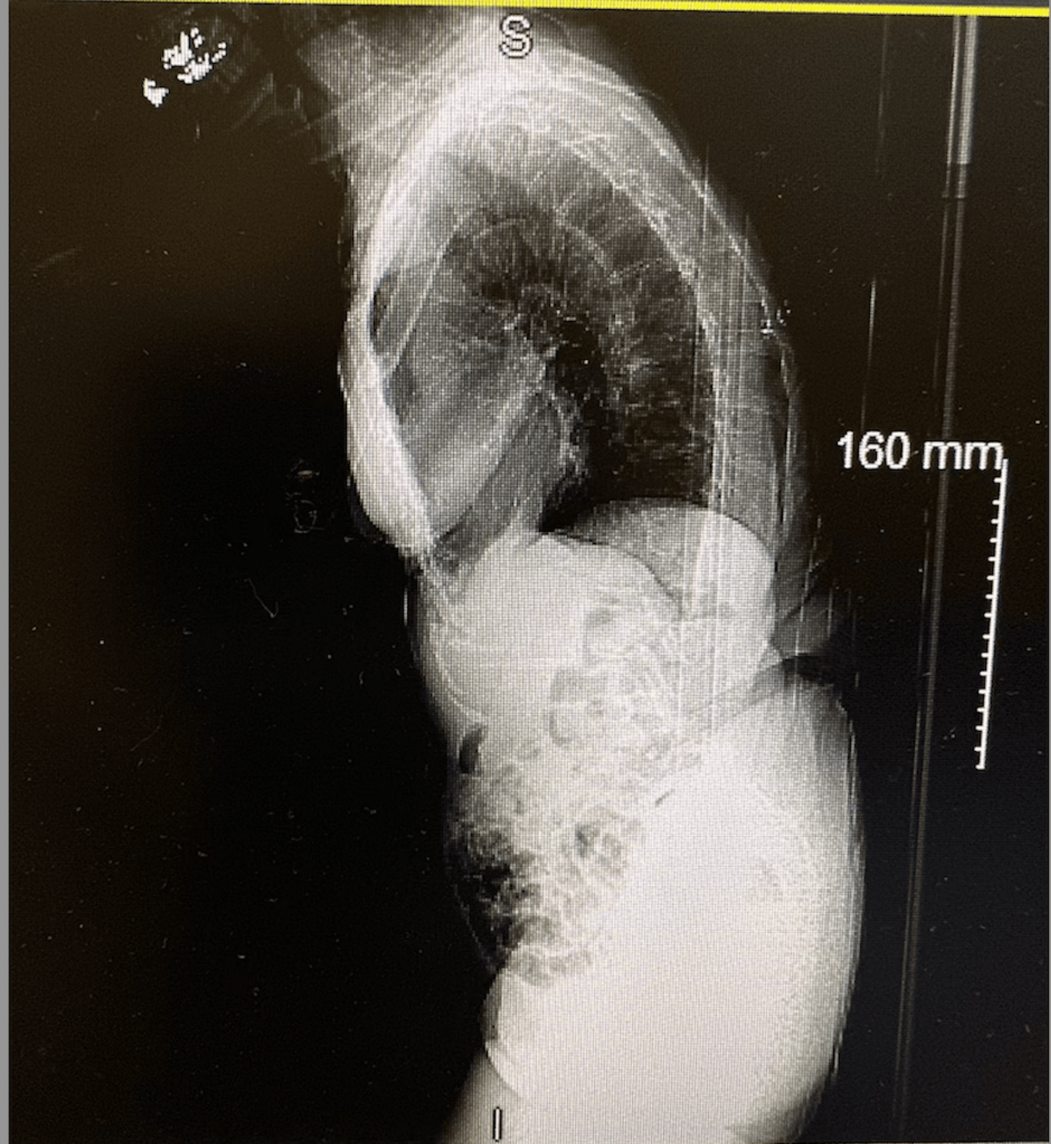
Lateral view CT scan of the thoracolumbar spine CT: computed tomography

A follow-up full-body computed tomography (CT) of the spine demonstrated findings of kyphoscoliosis with severe convex left lumbar and convex right upper thoracic spine curvature (Figures 2, 3). Multilevel, moderate disc height loss with minimal osteophytes in the anterior segments was present. No posterior spurring or compression of the cord was observed. Significant levoconvex lumbar scoliosis was observed, which limits the evaluation of the spine. There is a grade 1 anterior subluxation of L3 on L4 and a grade 2 anterior subluxation of L4 on L5. At the T12-L1 level, there is marked disc space narrowing and endplate sclerosis without significant spinal stenosis. The L1-2 level appears unremarkable. At L2-3, there is significant disc space narrowing, vacuum phenomena, posterior and right lateral osteophyte formation, resulting in mild central stenosis and right foraminal narrowing. Laminectomies are noted at L3-4 with associated vacuum changes and significant facet arthrosis, leading to moderate central stenosis and notable posterior impression on the thecal sac, more pronounced on the right. At L4-5, laminectomies are again seen with a prosthetic disc in place and facet arthrosis present, though no significant central stenosis is observed. The L5-S1 level shows marked disc space narrowing and vacuum phenomena without evidence of stenosis. Additionally, there are no acute abnormalities in the visualized retroperitoneum or bowel, but a right-sided pelvic kidney is noted as an anatomical variant.

Because of the severity of pain, structural changes in the spine, and failure to respond to medications and injections, a trial of SCS was discussed and fully explained to the patient and her husband. This included expectations, risks, benefits, device types, MRI compatibility, and the likelihood of improvement of function. An effective trial of SCS was performed on June 6, 2024. The patient experienced pain relief, with 70-80% pain reduction and full recovery of activities of daily living (ADLs) during the trial. Leads were removed in the clinic with ease without complication, and following her good response, arrangements were made for permanent implantation of the device.

She underwent implantation of a Vanta™ nonrechargeable SCS on July 15, 2024, in the right flank with no perioperative complications. The patient was brought to the operating room, placed in the prone position, and prepped and draped using sterile technique. After local anesthetic infiltration, a midline incision was made from L1 to L2. Using fluoroscopic guidance, two 14-gauge Tuohy needles were used to access the epidural space at the T12-L1 interlaminar level via the loss-of-resistance technique. Two eight-contact SCS leads were advanced into the posterior epidural space with the tips positioned at the top of the T8 vertebral body. Proper placement was confirmed on anterior-to-posterior (AP) and lateral fluoroscopic views. All placed electrodes were verified to provide appropriate coverage of the patient's area of pain.

Following lead placement, the patient was further sedated, and both leads were anchored with silk sutures. A right buttock pocket was created for the implantable pulse generator (IPG) using blunt dissection. The leads were tunneled to the IPG pocket and connected. Impedance testing confirmed that all contacts were functional. The IPG was placed into an antibiotic mesh (TYRYX envelope, Medtronic, Inc., Minneapolis, MN, USA) and inserted into the pocket with the label facing out. 

**Figure 4:**
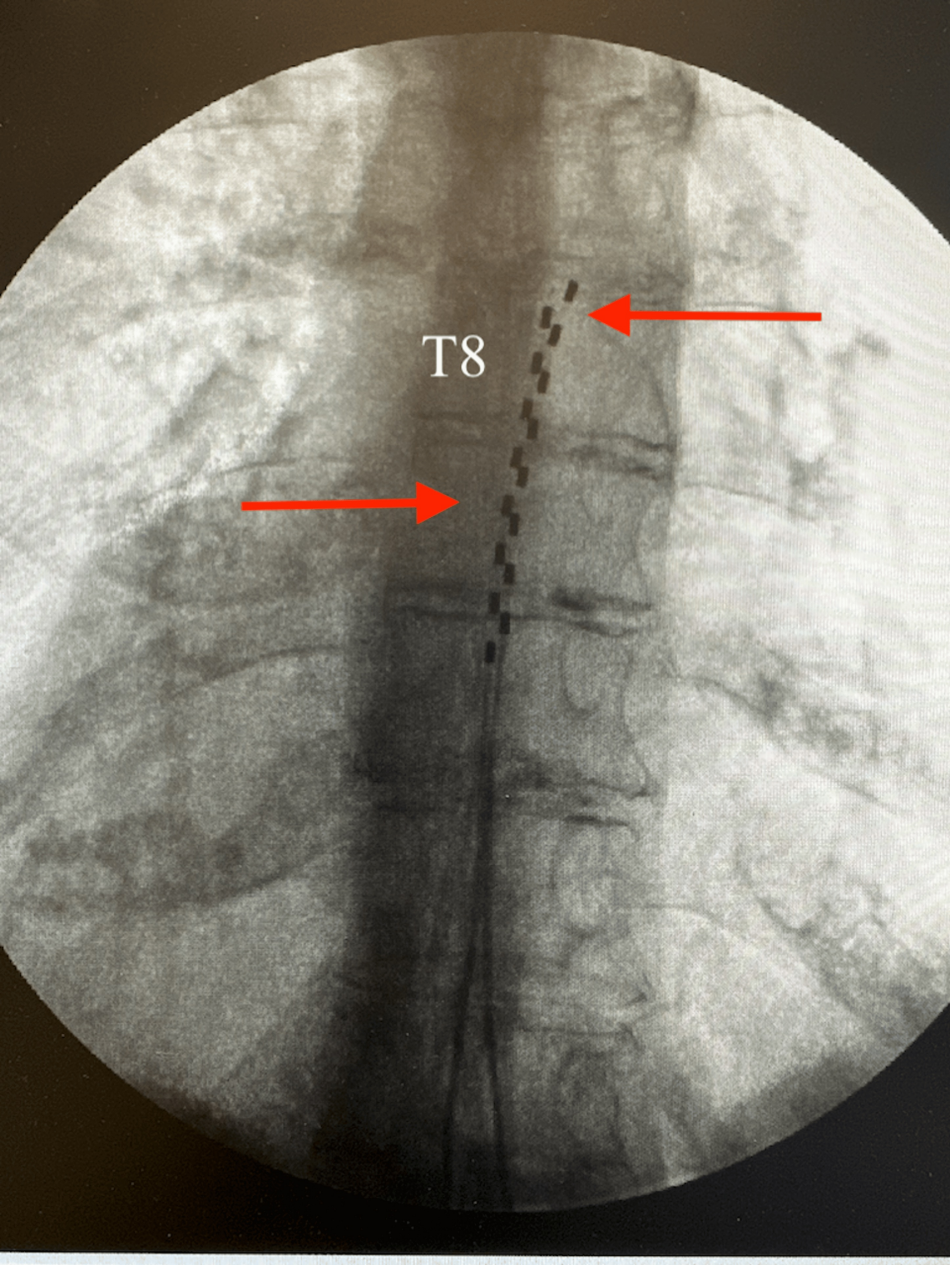
AP fluoroscopic view of the thoracolumbar spine showing spinal cord stimulator leads (red arrows) and the top of T8 labeled AP: anterior-to-posterior

**Figure 5:**
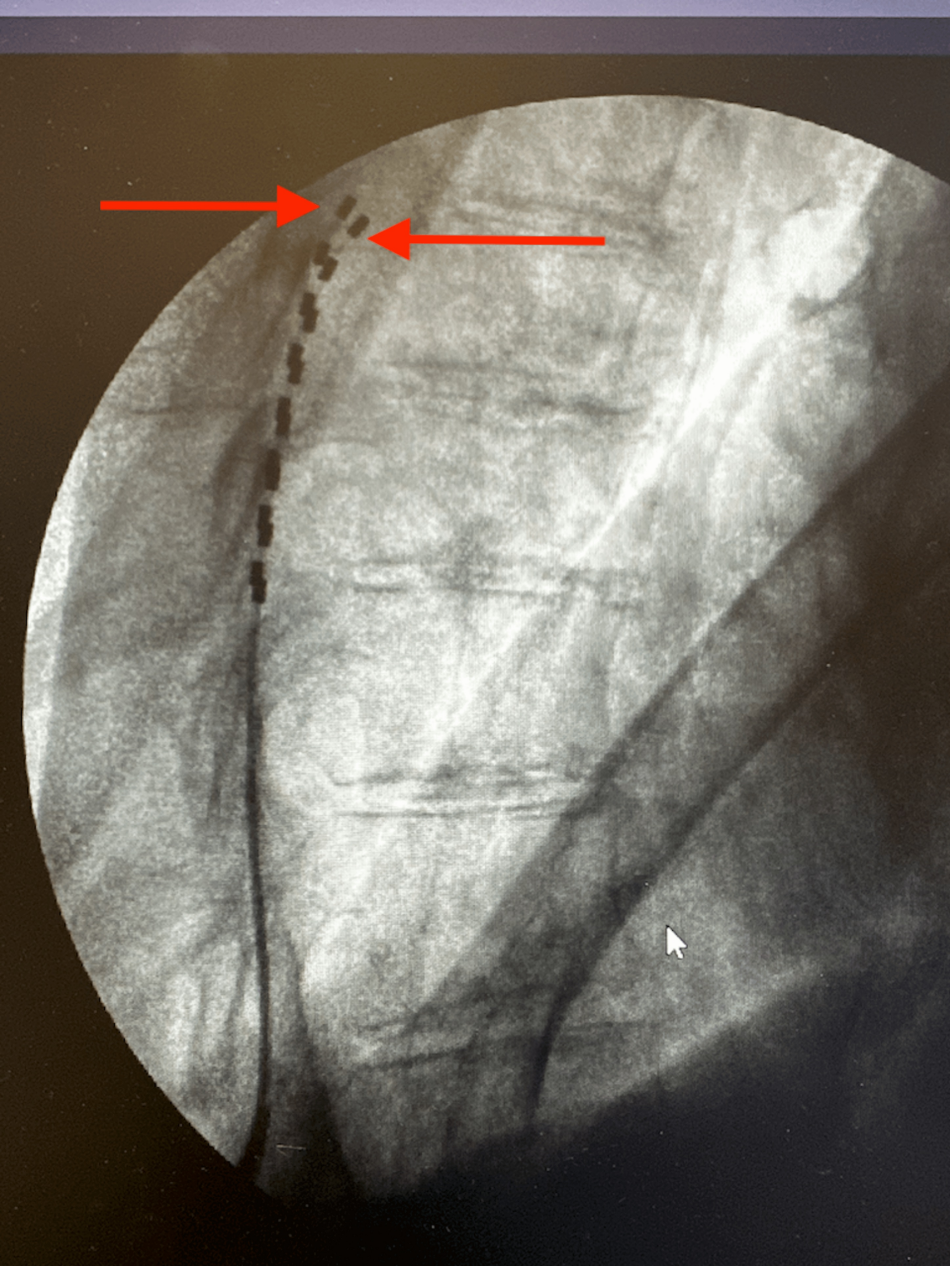
Lateral fluoroscopic view of the thoracolumbar spine showing stimulator leads (red arrows)

Both incisions were irrigated, treated with vancomycin powder, and closed in a layered fashion. Final AP and lateral fluoroscopy imaging confirmed stable lead positioning (Figures 4, 5). The patient tolerated the procedure well without complications and was discharged home in stable condition. Postop instructions were provided, with follow-up planned with the device representative in seven days and the clinic in two weeks for wound check and pain management. At her two-week follow-up, she noted continued pain relief of approximately 70-80% for her back and thighs with full restoration of ADLs. She returned to her baseline functional level, including the ability to walk upright without assistance. A follow-up two-month appointment showed the patient continued to have similar pain relief as her two-week follow-up.

This patient's SCS was selected for full-body MRI compatibility, and five programs (Table 1) were programmed with various amplitudes, pulse widths, and frequencies to optimize patient comfort and stimulation coverage.

**Table 1:** Spinal cord stimulation program settings overview Program 5 includes two subprograms (5A and 5B) to allow for alternating stimulation strategies. Subprogram A delivers higher amplitude and pulse width at 100 Hz for deeper or broader field stimulation. Subprogram B provides a lower-amplitude, lower-frequency alternative designed to reduce stimulation fatigue or address varying pain patterns

Program	Subprogram	Amplitude (mA)	Pulse width (µs)	Rate (Hz)
1		3.6	200	50
2		2.0	200	50
3		2.0	200	50
4		1.8	200	100
5	A	4.0	350	100
5	B	2.0	300	50

## Discussion

For adult patients with severe scoliosis, if conservative therapy failed, surgical intervention would have been the next step for a well-established approach for chronic back pain [4]. However, since this elderly patient has the presence of additional spinal pathology, including multilevel degenerative disc disease, facet arthropathy, prior instrumentation, and FBSS, surgical treatment would not be the best option for treatment. 

SCS is a minimally invasive procedure involving placing leads percutaneously in the epidural space of the spinal column [10]. The leads will stimulate large A-beta fibers (which carry touch and vibration), which can block pain signals from traveling from smaller C and A-delta fibers by “closing the gate” in the spinal cord's dorsal column [10]. This is according to the gate control theory of pain by Melzack and Wall, which will ultimately prevent pain from reaching the brain [10]. For the leads to be placed into the spinal cord, an epidural needle will be used to access the epidural space [10]. The relevant anatomy to be considered includes the vertebrae, ligaments (specifically the supraspinous ligament, interspinous ligament, then finally the ligamentum flavum), the spinal cord, arterial supply, and venous supply [10]. 

Preoperative preparation for SCS placement is usually imaging, with MRI being the most common [10]. In this case, detailed imaging of the epidural space and the anticipated lead placement site was crucial in navigating the patient’s complex spinal anatomy, including severe scoliosis, prior surgical changes, and multilevel degeneration. It allowed for precise procedural planning and helped correlate anatomical abnormalities with the patient’s pain distribution, ultimately contributing to the successful outcome despite significant structural challenges [10].

Trial stimulation is another pertinent preoperative measure for SCS. The trial requires one or more leads to be implanted and allows the patient to determine if their area of pain is covered and if they have an increase in 50% functionality and/or a 50% decrease in overall pain [10]. There has been debate as to whether SCS trials are needed [11,12].

An expert committee convened by the American Society of Regional Anesthesia and Pain Medicine developed 39 consensus-based recommendations to guide patient selection and trial use for SCS, emphasizing psychosocial screening and the value of trials in most cases [12]. While not definitive practice standards, these guidelines support individualized physician judgment and underscore that SCS trials, despite limitations, remain helpful in determining candidacy [12].

In contrast, a study by Eldabe et al. assigned thirty patients to a “Trial Group” (TG) and thirty-six patients to a “No Trial Group” (NTG). They determined that there was no difference in the superior outcome in the long-term when comparing the TG and NTG [13]. This study, along with many others, does not account for patients with spinal deformities such as this patient [11-13]. An SCS trial with this patient was able to demonstrate that SCS was able to provide immediate and dramatic relief, even with such distorted anatomy of the spine. Around 70-80% of her pain was gone with full recovery of her ADLs during the trial. More research will need to be conducted to demonstrate the impact of SCS trials on patients with anatomical challenges and possibly use SCS trials on a case-by-case basis for future patients. 

Most existing studies on SCS therapy are small, prospective, or retrospective in nature, resulting in a relative lack of established guidelines regarding contraindications for the procedure [4]. As with other elective procedures, standard contraindications apply. These include active infection at the surgical site, anatomical abnormalities that prevent safe lead placement, uncontrolled systemic illness, and bleeding diathesis that is not well managed [10]. The key one here is anatomical abnormalities, which have been stated to be a relative contraindication in several studies for SCS [3,4,10]. 

This patient’s spinal anatomy presented several features commonly cited as relative contraindications to SCS. She had severe kyphoscoliosis with convex left lumbar and convex right upper thoracic curvature and multilevel degenerative disc disease, causing tight interlaminar spacing and disc space narrowing. Such deformities can alter spinal dimensions, reduce epidural access, and complicate lead navigation and placement [3,4]. The Neuromodulation Appropriateness Consensus Committee and other expert sources identify spinal stenosis, scoliosis, and prior laminectomy as anatomic factors that may hinder successful implantation or stimulation coverage [3,4]. Additionally, the radiologic midline may differ from the physiologic midline in patients with spinal deformity, making it more difficult to achieve optimal lead placement using fluoroscopic guidance alone [3]. Meaning, the midline seen on imaging (like fluoroscopy) may not match the true functional midline of a patient’s nervous system. So, even if a lead looks perfectly centered on an X-ray, it might not align with the nerves in a way that provides optimal pain relief. Although broad paresthesia coverage and postimplant programming may provide partial compensation, accurate intraoperative lead placement is often crucial for long-term success, as seen with this patient. Preoperative planning should also account for IPG placement, considering factors such as cosmetic appearance, bony landmarks, body habitus, clothing preferences, and joint mobility [3]. 

Studies in patients with ASD have shown variable outcomes with SCS. These inconsistencies are often attributed to the extent and severity of spinal curvature, sagittal imbalance, altered epidural anatomy, and the presence of previous instrumentation or fusion [5,6]. In some cases, these deformities interfere with optimal lead positioning or limit the ability of stimulation to provide adequate coverage, leading to suboptimal clinical response [3,5,6]. As a result, surgical correction, such as osteotomy or long-segment fusion, is sometimes required for meaningful symptom relief [5,6].

However, this case challenges that trend. Despite significant scoliosis, kyphosis, and multilevel degeneration, the patient experienced 70-80% pain relief and full restoration of daily function following SCS implantation. This outcome demonstrates that, with careful preoperative planning and tailored intraoperative technique, SCS can still be a viable and effective treatment even in anatomically complex patients. It highlights the importance of individualized evaluation rather than excluding patients based solely on radiographic findings.

Elderly patients are often underrepresented in SCS trials due to concerns about frailty, comorbidities, and perceived lower success rates [7]. Outcomes of SCS in elderly patients remain mixed, with some studies demonstrating similar benefits to those seen in younger individuals, while others report diminished effectiveness in older populations [7-9]. In a retrospective study of 189 patients, those aged 65 and older showed comparable or greater improvements than younger patients in pain and affective measures following SCS, with significant gains in McGill Pain Questionnaire MPQ-sensory (p < 0.001) and MPQ-affective (p = 0.046) scores. These findings support the effectiveness of SCS in elderly populations, countering concerns about reduced benefit with age [8]. Another retrospective review of 174 patients found that age was a significant predictor of trial failure, with unsuccessful trial patients having a median age of 66 compared to 54 in those with successful trials (p = 0.005) [9]. Given that permanent implantation typically follows a successful trial, this finding suggests that older patients may be less likely to receive an implant, potentially contributing to their underrepresentation in SCS cohorts and perceived lower success rates. These concerns may lead to clinical hesitation and underutilization of SCS in older adults.

This case presentation adds to the growing body of evidence supporting the safe and effective use of SCS in appropriately selected elderly patients. Despite her advanced age and complex spinal anatomy, this 78-year-old woman experienced 70-80% pain relief and full restoration of ADLs. Her outcome highlights the importance of individualized assessment, careful planning, and clinical judgment in extending the benefits of neuromodulation to older adults who are often overlooked for this therapy.

The success of this case also raises some thoughtful questions regarding common clinical practices and research protocols that customarily exclude patients with spinal deformity or prior fusion from SCS trials. While strict inclusion criteria ensure internal validity in randomized controlled studies, they may not fully represent the actual population of patients in their complexity. This case suggests that relative contraindications to SCS, such as severe scoliosis, prior lumbar fusion, and advanced age, should be interpreted with greater flexibility, as even anatomically complex patients may still achieve excellent outcomes with appropriate planning and technique.

This case suggests that with proper patient selection and technique, patients with complicated spinal anatomy and older patients should be carefully looked at before excluding them from neuromodulation therapies. Future studies should establish the efficacy of SCS in these underrepresented groups using larger-scale trial designs. In the short term, clinicians can be encouraged to use their own judgment in assessing SCS candidacy and not adhere overly strictly to anatomical exclusion criteria.

## Conclusions

This case demonstrates that SCS can be a safe and effective treatment for select elderly patients with complex spinal anatomy, including severe scoliosis and prior fusion. While advanced age is not a contraindication to SCS, the procedure is less commonly pursued in elderly patients due to concerns about frailty, comorbidities, and surgical risk. Moreover, existing literature suggests that older patients are less likely to be offered SCS in the first place, potentially limiting access to a therapy that could significantly improve their quality of life. This case challenges the traditional hesitancy to consider neuromodulation in such populations, showing that with careful preoperative planning and individualized care, SCS remains a viable option to restore function and well-being in patients who are often overlooked for this therapy.
